# Polycystic ovary syndrome and autism: A test of the prenatal sex steroid theory

**DOI:** 10.1038/s41398-018-0186-7

**Published:** 2018-08-01

**Authors:** Adriana Cherskov, Alexa Pohl, Carrie Allison, Heping Zhang, Rupert A. Payne, Simon Baron-Cohen

**Affiliations:** 1 Autism Research Centre, Department of Psychiatry, University of Cambridge, Douglas House, 18B Trumpington Road, Cambridge, CB2 8AH UK; 2 Department of Biostatistics, Yale University School of Public Health, New Haven, CT 06520 USA; 3 Primary Care Unit, Institute of Public Health, University of Cambridge, Cambridge, CB2 OSR UK; 4 Centre for Academic Primary Care, Bristol Medical School, University of Bristol, Bristol, BS8 2PS UK; 5 CLASS Clinic, Cambridgeshire and Peterborough NHS Foundation Trust, Cambridge, UK

## Abstract

Elevated levels of prenatal testosterone may increase the risk for autism spectrum conditions (autism). Given that polycystic ovary syndrome (PCOS) is also associated with elevated prenatal testosterone and its precursor sex steroids, a hypothesis from the prenatal sex steroid theory is that women with PCOS should have elevated autistic traits and a higher rate of autism among their children. Using electronic health records obtained from the Clinical Practice Research Datalink (CPRD) in the UK between 1990 and 2014, we conducted three matched case-control studies. Studies 1 and 2 examined the risk of PCOS in women with autism (*n* = 971) and the risk of autism in women with PCOS (*n* = 26,263), respectively, compared with matched controls. Study 3 examined the odds ratio (OR) of autism in first-born children of women with PCOS (*n* = 8588), matched to 41,127 controls. In Studies 1 and 2 we found increased prevalence of PCOS in women with autism (2.3% vs. 1.1%; unadjusted OR: 2.01, 95% CI: 1.22–3.30) and elevated rates of autism in women with PCOS (0.17% vs. 0.09%, unadjusted OR: 1.94 CI: 1.37–2.76). In Study 3 we found the odds of having a child with autism were significantly increased, even after adjustment for maternal psychiatric diagnoses, obstetric complications, and maternal metabolic conditions (unadjusted OR: 1.60, 95% CI: 1.28–2.00; adjusted OR: 1.35, 95% CI: 1.06–1.73). These studies provide further evidence that women with PCOS and their children have a greater risk of autism.

## Introduction

Autism spectrum conditions (henceforth autism) are characterised by impairments in communication and social interaction, alongside unusually narrow interests and repetitive behaviours. The prenatal sex steroid theory of autism posits that among the many players likely implicated in autism are steroid sex hormones and androgens in particular. Autism is disproportionately observed in males, with up to four times as many males diagnosed with the condition^1,2^. In addition, the hallmarks of autism suggest an exaggeration of systemising and down-regulation of empathy^1,3^. Since these psychological characteristics are associated with prenatal testosterone^4,5^, elevated exposure to prenatal testosterone during a critical stage of neurological sex differentiation may also be contributing to the male bias of autism.

Elevated levels of amniotic steroid hormones, including androgens, progesterone, 17a-hydroxy-progesterone, and cortisol, have been associated with autism in both male and female children^6^. Furthermore, prenatal testosterone levels in amniotic fluid in the second trimester, within the hypothesised critical period for neural sexual differentiation (8–24 weeks of gestation)^7,8^, positively correlated with attention to detail^9^, interest in systems^4^, narrow interests^10^, and other specific autistic traits^11,12^. Lombardo et al. have shown that variation in foetal testosterone predicts grey matter volume in three sexually dimorphic brain regions (planum temporale, a language area, superior temporal sulcus, involved in face-processing, and temporo-parietal junction, involved in theory-of-mind), all of which are atypical in structure and/or function in autism^13^. Together, these findings have come to define the prenatal sex steroid theory of autism.

An increased prevalence of androgen-related conditions, such as polycystic ovary syndrome (PCOS) has also been reported in women with autism^14–16^. One of the most common endocrine disorders, PCOS occurs in 7–10% of women of reproductive age^17^. PCOS is defined by hyperandrogenism with ovulatory dysfunction and/or ovarian cysts (polycystic ovaries or PCO). The independent association of prenatal androgens with PCOS suggests predictions from the prenatal sex steroid theory of autism: women with PCOS should have a higher rate of autistic traits and a higher rate of autism among their children.

Studies investigating the relationship between autism and PCOS have fallen into two categories: those that investigated steroidogenic traits in women with autism, and those that investigated autistic traits in women with PCOS. In the first category, self-report of reproductive and sex-steroid-related symptoms suggests an increase of these symptoms in women with autism^15,16^. Women with autism also have increased levels of androstenedione (an adrenal androgen)^18^, elevated levels of luteinizing hormone (LH), and an increased free androgen index (FAI) compared to typical controls^19^. In the second category, women with PCOS score higher on measures of autistic traits^20^, as do their daughters, but not their sons^21^. A recent population study in Sweden found a significantly increased risk of autism in children of mothers with PCOS, which was exacerbated by comorbid maternal metabolic syndrome^22^. Here, we contribute to both approaches, presenting a comprehensive picture of autism and PCOS association in women and their children using the Clinical Practice Research Datalink (CPRD) electronic health record database.

## Methods

### Data source

Anonymized electronic medical records were obtained from the UK-based primary care CPRD database, which has been collecting anonymized patient data from the NHS since 1988^23^. These records contain detailed clinical diagnoses for over 15 million patients in the UK, entered and updated by each patient’s general practitioner (GP) using the Read code system. Virtually all UK residents have a registered GP, who serves not only as their primary care physician but as a gatekeeper to National Health Service (NHS) specialist care. As a result, GP records provide a comprehensive clinical summary even when diagnoses are made by specialists, and the CPRD dataset is generally representative of the UK population. CPRD also provides a Mother–Baby Link to match mothers and children, which has been previously validated^24,25^. Ethical approval was granted by the CPRD Independent Scientific Advisory Committee (Protocol 14_224R).

### Study design and population

Three separate matched case-control studies were conducted. In Study 1, we examined the risk of PCOS in women with autism compared to those without autism. In Study 2, we examined the risk of autism in women with PCOS compared to those without PCOS. In Study 3, we examined the risk of autism in first-born children of mothers with PCOS compared to first-born children of mothers without PCOS; mothers of cases were drawn from the cohort defined for Study 2.

**Fig. 1:**
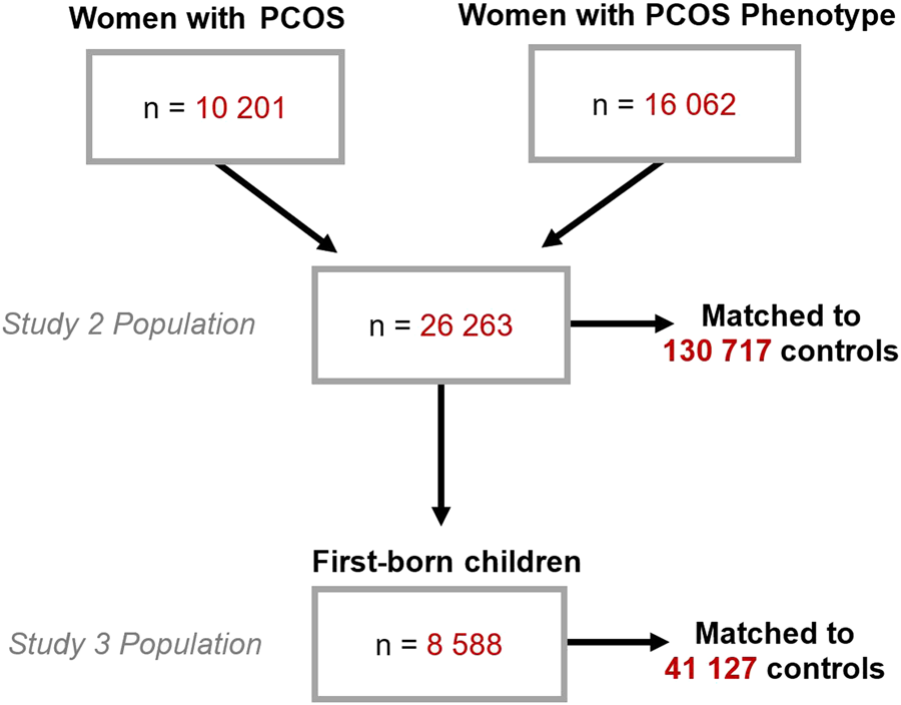
Cohort inclusion for women with PCOS and their children

The population was restricted to females registered in the CPRD between January 1st, 1990 and December 31st, 2014, with at least 3 months CPRD medical history prior to PCOS or autism diagnosis (to maximise data quality) and aged 21 years or older at the end of the study period. These characteristics, in addition to diagnoses of inclusion, were specified to extract our case and control populations for each study from the database. *n* = 971 women diagnosed with autism were included in Study 1. *n* = 26,263 women with PCOS phenotype (discussed below in section “Exposure, outcomes, and covariates”) were included in Study 2. For Study 3, the CPRD Mother–Baby Link was used to identify first-born children from mothers included in the Study 2 PCOS population (Fig. 1). The Study 3 population included *n* = 8,588 children of women with the PCOS phenotype in Study 2 who had first-born children linked in the database.

For Studies 1 and 2, we identified all matched controls for each case based on year of birth and GP practice and then randomly drew up to five or included all if fewer than five were available (Fig. 1). Study 1 included 4,855 controls and Study 2 included 130,717 controls. The same methods applied in Study 3, only controls were selected from children of mothers without a diagnosis of PCOS, and were matched on gender, GP practice, and birth year within 2 years of cases. Study 3 included 41,127 children and their mothers (*n* = 41,127) as controls. We adjusted for maternal age at childbirth in subsequent statistical modelling.

### Exposure, outcomes, and covariates

UK general practice records coded diagnostic information using the Read code clinical system. PCOS was defined by Read codes for polycystic ovarian syndrome and Stein–Leventhal syndrome. We also included women with codes consistent with the Rotterdam definition of PCOS, despite absence of a formal PCOS diagnosis. This PCOS phenotype thus included women with PCOS Read codes, as well as women with a clinical code for PCO and code for either hyperandrogenemia or menstrual irregularities. Because test results are not always consistently reported in the CPRD, we included any women with any record of having a test of hyperandrogenemia, whether or not it was abnormal. Patients with diagnoses of Cushing syndrome, adrenogenital disorders, or congenital adrenal hyperplasia were excluded. As secondary analyses, we considered the three key diagnostic components of PCOS (ovulatory dysfunction, hyperandrogenism, PCO) separately, and as part of the NIH diagnostic criteria (hyperandrogenism plus ovulatory dysfunction), and Rotterdam criteria (two out of three components) with the exclusion of the aforementioned endocrine disorders. In our examination of these diagnostic components, our criteria of hyperandrogenemia (and thus inclusion in NIH or Rotterdam criteria) includes only women with abnormal test results (see Supplementary materials).

Diagnosis of autism in both women and children was based on revised Read code lists previously defined by Smeeth et al.^26^. Previous studies have confirmed the validity of CPRD autism diagnoses (where diagnosis of a pervasive developmental disorder (PDD), was confirmed in 92.5% of cases), and thus Read codes have good positive predictive value for diagnosis^26^.

Diagnostic sub-categories of autism according to historical diagnostic methods (e.g. Asperger’s syndrome, PDD) were examined for Studies 1 and 2. The lifetime rates and risk of depression, anxiety, schizophrenia-like mental illness, diabetes mellitus (type 1 and 2), obesity, as well as PCOS characteristics, such as menstrual irregularities, hyperandrogenemia, and PCO until the end of the study period were also examined. In Study 3, the aforementioned covariates, in addition to diagnoses of infertility, pre-eclampsia, and complications in childbirth for the included child, and maternal gestational diabetes for the included child were compared between mothers of cases and controls. All covariates included were based on Read code lists defined for this study (Supplementary materials).

### Statistical analysis

Differences in case-control sample characteristic data were tested with two-tailed independent sample *t*-test for continuous variables and chi-square test for categorical variables. The association of PCOS, autism, and covariates was determined by calculating odds ratios (ORs) and 95% confidence intervals by conditional logistic regression. An adjusted conditional regression model in Studies 1 and 2 included comorbid psychiatric conditions (lifetime history of depression, anxiety, and schizophrenia-related illness). In Study 3, two models were used in conditional logistic regression analysis. Model 1 included adjustment for maternal age at childbirth, depression, anxiety, and schizophrenia-related illness. Model 2 additionally adjusted for maternal lifetime diagnosis of obesity, diabetes, complications during childbirth for the included child, and gestational diabetes for the included child. G*Power 3.1 was used to determine covariate number to ascertain power with our population size^27^. All statistical analyses were performed in SAS 9.4 (SAS Institute Inc., Cary, NC, USA) and RStudio Version 3.2.0 using the survival package^28,29^.

## Results

### Study 1

A significantly higher percentage of women with autism were diagnosed with PCOS than controls (Table 1). Women with autism were also more likely to have any of the three core clinical signs/symptoms of PCOS (Table 1). Diabetes was more prevalent in the autism group compared with controls, as were depression, anxiety, and schizophrenia-related conditions (Supplementary Table 1). Adjustment for comorbid psychiatric conditions preserved the trend for increased odds of PCOS in women with autism, although this was not significant in the PCOS Read code population (Table 1).

**Table 1:** Characteristics and OR for women with ASC in the CPRD

Characteristics	ASC		Controls		Controls–ASC	
	Count	% Total	Count	% Total	Unadjusted OR (95% CI)	Adjusted OR^a^ (95% CI)
Total	971	–	4855	–	--	–
Mean age	30.3 ± 9.1	–	30.3 ± 9.1	–	–	–
Mean age at ASC diagnosis	21.1 ± 12	–	–	–	–	–
Mean age at PCOS diagnosis	23.7 ± 6.0	–	26.2 ± 8.7*	–	–	–
*PCOS*						
PCOS Read code	22	2.3	55	1.1	2.01 (1.22–3.30)**	1.31 (0.72–2.39)
PCOS (NIH criteria)	72	7.4	150	3.1	2.50 (1.87**–**3.35)*	1.65 (1.17–2.32)**
PCOS (Rotterdam criteria)	76	7.8	171	3.5	2.33 (1.76**–**3.08)*	1.57 (1.13–2.18)**
Anovulation	150	15.4	443	9.1	1.82 (1.61**–**2.34)*	1.42 (1.13–1.78)**
Hyperandrogenemia	193	19.9	604	12.4	1.75 (1.46**–**2.09)*	1.44 (1.18–1.75)**
Polycystic ovaries	55	5.7	75	1.5	3.83 (2.68**–**5.46)*	1.00 (0.58–1.73)

^a^Adjusted for comorbid psychiatric conditions (anxiety, schizophrenia-related illness, depression)

*p-value < 0.001; **p-value < 0.01

### Study 2

Autism was almost two times more prevalent in PCOS cases (*n* = 26,263) than in controls (*n* = 130,717). Compared with controls, PCOS cases also had an increased prevalence of diabetes and obesity (Table 2). PCOS cases were also more likely to have depression, anxiety, and schizophrenia-related conditions, which persisted with adjustment for comorbid psychiatric conditions (Table 2). Adjustment for these psychiatric conditions in the model attenuated the increased odds for autism in PCOS cases, although the trend was still present.

**Table 2:** Characteristics and OR for women with PCOS in the CPRD

Characteristics	PCOS phenotype^a^		Controls		Controls**–**PCOS phenotype	
	Count	% Total	Count	% Total	Unadjusted OR (95% CI)	Adjusted OR (95% CI)^b^
Total	26,263	–	130,717	–	–	–
Mean age	35.5 ± 8.5	–	34.5 ± 8.5	–	–	–
Mean age at PCOS diagnosis	27.6 ± 7.5	–	–	–	–	–
Mean age at ASC diagnosis	16.2 ± 8.9	–	18.1 ± 11.8	–	–	–
*ASC*						
Autism	25	0.10	62	0.05	2.01 (1.26**–**3.20)*	–
Asperger’s	19	0.07	46	0.04	2.06 (1.21**–**3.51)*	–
PDD-NOS	1	0.00	7	0.00	–	–
Total	45	0.17	115	0.09	1.94 (1.37–2.76)**	1.41 (0.96–2.07)^
*Other psychiatric conditions*						
Depression	8700	33.1	22,247	17.0	2.53 (2.45**–**2.61)**	2.16 (2.09–2.24)**
Anxiety	5608	21.4	13,825	10.6	2.37 (2.28**–**2.45)**	1.76 (1.69–1.84)**
Schizophrenia	233	0.89	696	0.53	1.67 (1.44–1.94)**	1.11 (0.93–1.32)
*Metabolic conditions*						
Diabetes mellitus	898	3.4	1218	0.9	3.87 (3.54–4.23)**	–
*Obesity*	4904	18.7	3976	3.0	7.80 (7.44–8.18)**	–

^a^Read code diagnosis of PCOS or symptoms/signs consistent with the PCOS phenotype

^b^Adjusted for comorbid psychiatric conditions (anxiety, schizophrenia-related illness, depression)

*p-value < 0.01; **p-value < 0.001; ^*p*-value = 0.084

10,201 of the cases had a diagnostic code for PCOS, and rates of autism were significantly higher than for cases with a PCOS phenotype (Supplementary Table 3). Rates of ovulatory dysfunction and hyperandrogenism were greater in cases with a PCOS phenotype than PCOS diagnosis, although the age of diagnosis was significantly lower for PCOS cases (Supplementary Table 3). Although no controls had a coded diagnosis of PCOS, a small percentage of controls may have undiagnosed PCOS (2.8–3% according to retrospective NIH and Rotterdam criteria, Supplementary Table 4). Prevalence of diabetes and the aforementioned psychiatric diagnoses were not significantly different between those cases with and without a diagnostic code for PCOS, although rates of obesity were significantly lower (Supplementary Table 3).

### Study 3

Of the 26,263 cases with a PCOS phenotype in the Study 2 population, 8,588 mothers had first-born children registered in the CPRD database that were included for analysis. The prevalence of metabolic conditions, pregnancy complications, and psychiatric conditions used as covariates in further models for analysis differed significantly between cases and controls (Table 3). Mothers with PCOS also had a significant increase in obesity and gestational diabetes compared to mothers with the PCOS phenotype (Supplementary Table 5).

**Table 3:** Characteristics of PCOS mothers and matched controls in the CPRD

Maternal characteristics	PCOS phenotype		Controls		*p*-value^a^
	Count	% Total	Count	% Total	
Total	8588	–	41127	–	–
Mean maternal age	37.8 ± 7.2	–	38.1 ± 7.2	–	<0.001
Mean maternal age at childbirth	27.7 ± 5.2	–	27.9 ± 5.5	–	<0.001
*Metabolic conditions*					
DM Type 1 and 2 (lifetime)	298	3.5	458	1.1	<0.001
Obesity (lifetime)	1686	19.6	2245	5.4	<0.001
DM Type 1 and 2 (before pregnancy)	98	1.1	166	0.4	<0.001
Obesity (before or during pregnancy)	678	7.9	719	1.7	<0.001
*PCOS characteristics (lifetime)*					
Rotterdam	5890	68.6	2203	5.4	<0.001
NIH	2893	33.7	2096	5.1	<0.001
Ovulation	5513	64.2	7945	19.3	<0.001
Hyperandrogenemia	3173	37.0	6887	16.7	<0.001
PCO	6698	78	662	1.6	<0.001
*PCOS characteristics (before pregnancy)*					
Ovulation	2974	34.6	3139	7.6	<0.001
Hyperandrogenemia	1515	17.6	3776	9.2	<0.001
PCO	3487	40.6	538	1.3	<0.001
*Pregnancy complications*					
Childbirth complications (this pregnancy)	68	0.8	208	0.5	0.002
Gestational diabetes (this pregnancy)	226	2.6	385	0.9	<0.001
Infertility diagnosis	2062	24.0	2459	6.0	<0.001
Pre-eclampsia (this pregnancy)	1	0.01	6	0.01	–
*Psychiatric conditions (lifetime)*					
Depression	3528	41.1	12565	30.6	<0.001
Anxiety disorders	2058	24.0	7188	17.5	<0.001
Schizophrenia and mental illness	54	0.6	187	0.5	0.04
*ASC*	4	0.05	7	0.02	0.20

*DM* = diabetes mellitus

^a^*p*-values for two-tailed Student’s *t*-test or Chi-square test for PCOS phenotype compared with controls are shown

In the unadjusted model, mothers with PCOS had higher odds of having a child with autism than did controls (Table 4). Although the effect size was attenuated, the association persisted when adjustment was made for maternal age at childbirth, history of maternal depression and anxiety, and in Model 2, additionally for endocrine-related disorders including obesity, gestational diabetes, and infertility. Finally, stratification into male and female children did not reveal a significant difference in autism risk (Table 4).

**Table 4:** ASC OR in first-born children of women with PCOS in the CPRD

	PCOS			Control			Unadjusted	Model 1	Model 2
	ASC	Total	Prevalence (%)	ASC	Total	Prevalence (%)	OR (95% CI)	OR (95% CI)	OR (95% CI)
Total	104	8588	1.21	314	41127	0.76	1.60 (1.28–2.00)*	1.48 (1.18–1.87)*	1.35 (1.06–1.73)**
Male	90	4417	2.04	267	21098	1.27	1.63 (1.28**–**2.08)*	1.53 (1.20**–**1.97)*	1.42 (1.09**–**1.84)***
Female	14	4171	0.34	47	20029	0.23	1.43 (0.79**–**2.60)	1.21 (0.64**–**2.25)	1.05 (0.54**–**2.06)

Model 1 = adjusted for maternal age (at childbirth), depression, anxiety, schizophrenia-related illness

Model 2 = adjusted for maternal age (at childbirth), depression, anxiety, schizophrenia-related illness, complications at childbirth, obesity, diabetes, and gestational diabetes

*p-value < 0.001; **p-value < 0.05; ***p-value < 0.01

## Discussion

This is the first study to our knowledge in the UK examining the association of PCOS and autism, as well as the first population-based study examining the prevalence of PCOS in women with autism. We showed that in the UK, women with autism have an approximately two-fold increase in risk for PCOS, and women with PCOS also had a two-fold increase in rates of autism. Finally, women with PCOS had 35% increased odds of having a first-born child with autism, after adjusting for comorbid maternal psychiatric diagnosis, metabolic conditions, and complications in childbirth.

This study independently confirms a recent population study in Sweden that found women with PCOS had 56% increased odds of having a child with autism^22^. A previous study by Palomba et al. found that autistic traits are increased in children of women with PCOS and are significant (*p* < 0.05) among daughters but not sons^30^. However, like Kosidou et al., we also did not observe increased risk in females, though our small sample size of female cases warrants further study. Additional research will be important, as gender difference in risk may inform the aetiological model. Our study also confirms the results of Cesta et al., who recently found increased odds (unadjusted OR: 2.09 CI: 1.79–2.44, OR adjusted for comorbid psychiatric conditions: 1.55 CI: 1.32–1.81) of having autism in women with PCOS, although our adjusted model did not achieve significance due to the smaller sample size^31^.

Our findings indicate a common process may be contributing to the development of autism and PCOS. Increased steroidogenic activity is one key common denominator. The association with prenatal steroidopathies and autism was discussed earlier as the foundation of the prenatal sex steroid hypothesis. These findings can be linked to biological mechanisms, as structural influences of prenatal androgens and other steroid hormones are extensive in the mammalian prenatal brain and several morphological differences have been correlated with prenatal sex steroid influences^32–34^. At the cellular level, sex steroids act on a variety of developmental processes including selective cell death^35^, synaptogenesis and synaptic recruitment^36^, axon growth^37^, neurogenesis, and the pruning of synaptic spines^38^. Prenatal steroid hormones also have extensive epigenetic effects on a wide range of genes that may lead to the long-lasting organisational effects of prenatal sex steroids^39^.

Foetal exposure to high levels of androgens in utero is also sufficient to trigger the epigenetic, developmental, and physiological hallmarks of PCOS in animal models^40,41^. In humans, both foetal and maternal androgen excess has been implicated in PCOS^42–44^, and daughters of women with PCOS have significantly increased pre-pubertal hyperinsulinemia and biochemical hyperandrogenism in late puberty^45^. Women with PCOS also had even greater hyperandrogenemia during pregnancy, especially in the 2nd and 3rd trimesters^46–48^. Placentas of women with PCOS showed increased steroidogenic activity and decreased p450 aromatase activity, which is responsible for the breakdown of androgens, suggesting a mechanism of androgen accumulation^47^. Thus, women with PCOS may have higher levels of androgens in pregnancy, and prenatal androgens are implicated in the development of PCOS.

Steroid dysregulation leading to hyperandrogenism in both disorders is likely due to genetic, epigenetic, and environmental causes acting in concert. A genetic component may underlie our observed association between autism and PCOS. However, this genetic component likely still involves steroidogenesis, as several overlapping genes associated with both PCOS and autism have been associated with steroid synthesis. *CYP19A*, for instance, involved in aromatising testosterone to estradiol has been associated with PCOS pathogenesis^49^ and risk^50,51^; it has also been associated with autism^52^. A similar enzyme, *CYP17A*, involved in key catalytic steps in androstenedione synthesis has been consistently implicated in theca cell androgen overproduction, a key feature of PCOS and has been associated with autism risk^52^.

Hyperinsulinemia is a factor closely related to increased steroidogenic activity. Our work shows 3.5-fold and 3.9-fold increased odds of developing diabetes mellitus (type 1 and 2) in women with autism and PCOS, respectively, suggesting a close association of both with insulin and insulin resistance. Insulin plays a significant role in the pathogenesis of PCOS, maintenance of hyperandrogenemia, and abnormal placental and foetal steroidogenesis^50^. Chronic foetal exposure to elevated glucose will result in hyperinsulinemia and downstream hormonal effects including foetal hyperandrogenemia^53,54^. The placental aromatase-deficiency in PCOS described earlier may also be further exacerbated by gestational diabetes and hyperinsulinemia^55^, which are more common in women with PCOS^56,57^. If placental aromatase is compromised, maternal testosterone may be able to cross the placenta^55^. Examining the association of maternal gestational diabetes and autism is an important target for future research.

We also demonstrated an association between obesity and both autism and PCOS. Obesity has been linked to PCOS outcomes, and overweight and obese women have greater rates of hyperandrogenemia, likely resulting from obesity-induced hyperinsulinemia^58^. Hyperinsulinemia stimulates ovarian androgen production and decreases sex-hormone-binding globulin. Obesity may also specifically exacerbate sex-steroid dysregulation during pregnancy in women with PCOS. High triglycerides and low adiponectin have been associated with hyperinsulinemia during pregnancy^59^. Another study found significantly increased odds of obese mothers having a child with autism^49^, while Kosidou et al. demonstrated an increasing trend in autism risk with severity of maternal metabolic condition and obesity^22^. Although it is particularly difficult to tease apart obesity, hyperinsulinemia, and hyperandrogenemia, given they are so intricately intertwined, the association of all three with both autism and PCOS increases the likelihood of a related mechanism.

Our findings may indicate that severity of PCOS is also associated with greater risk for autism in women. We found a significant increase in prevalence and decreasing trend for age of autism diagnosis in our PCOS case group defined uniquely by PCOS Read codes, compared to the PCOS phenotype group that included cases additionally diagnosed by Rotterdam criteria. We observed a significantly decreased age of diagnosis of hyperandrogenemia, menstrual irregularities, and obesity, all of which contribute to PCOS severity. Furthermore, women who do not seek treatment for PCOS may not receive a diagnosis and be documented in the CPRD. As a result, symptom severity may contribute to increased risk of autism. Future research should examine the correlation between risk of autism and the severity of hyperandrogenemia in women with PCOS.

In conclusion, here, we explain our results in light of the hypothesis that prenatal hyperandrogenism and steroid dysregulation are key players in the development of both autism and PCOS. Since the symptoms of PCOS include hyperandrogenemia in pregnancy, PCOS itself is sufficient to trigger pathways involved in the development of autism. According to this hypothesis then, we also observe PCOS and steroidopathies in women with autism, because PCOS and metabolic dysregulation are related to prenatal steroid excess. The cause of this prenatal steroid excess may be genetic or may involve a positive feedback loop with maternal hyperinsulinemia or hyperandrogenemia. Future studies examining the quantitative association between biochemical hyperandrogenism, hyperinsulinemia, and autism, and whether hyperandrogenism is foetal, maternal, or placental, will be important avenues for future research.

This study also found that women with both autism and PCOS were at risk for a range of other psychiatric conditions. It has been shown that 70% of children with autism were diagnosed with another psychiatric disorder, including depression and anxiety disorders^60,61^. Our study confirms these findings, as we find significantly increased odds of anxiety, depression, as well as schizophrenia-related mental illness in women with autism.

Women with PCOS were also at risk for other psychiatric conditions. Rates of depression and anxiety were elevated in women with PCOS (33.1% and 21.4%, vs. 17.0% and 10.6% in controls, respectively). Other studies have found similar associations between PCOS and depression and anxiety^62–65^, and report that 40% of women with PCOS experienced clinical depression^64^, and 34% of women with PCOS were diagnosed with anxiety^62^. Our observed prevalence was higher than that found in the Swedish population (16.4% vs. 11.1% in PCOS cases vs. controls)^31^. This difference may be due to different medical code lists and definitions, as we included, for instance, bipolar and unipolar depression in one category. Interestingly, they associated increased prevalence of psychiatric conditions with hyperandrogenemia, and found increased risk for psychiatric diagnoses in unaffected siblings, suggesting the association may not only be caused by the stresses of having PCOS^31^. Nevertheless, the role of androgens in depression remains controversial, and several other studies have failed to find an association beyond a psychological one^66,67^. The underlying mechanisms for prevalence of mood disorders in women with PCOS has yet to be elucidated.

Although we present a comprehensive association between PCOS and autism diagnoses in women and their children, we acknowledge limitations of this study. Only first-born children of women with PCOS were included to ascertain independence. As studies have shown decreased prevalence of autism in first-born children^68^, this may be one reason we underestimate the rates of autism in children in the general population. As a result of missing information recorded in the CPRD, we did not control for marital status, alcohol-use, specific hormone or infertility treatments, and socioeconomic background (i.e. income, education, occupation). Furthermore, the Mother–Baby Link lacked information regarding possible paternal influences, including age or psychiatric history. Finally, the Mother–Baby Link requires continuous registration with GPs, which may limit how many children are available for follow-up for autism diagnosis and may bias results towards earlier diagnoses.

The relatively low proportion of women diagnosed with PCOS in our study is also surprising, considering prevalence of PCOS has been estimated at 6–10% according to NIH criteria, and almost 20% according to Rotterdam criteria^69^. As discussed previously, this may be due to reporting based on symptom severity but may also be due to underreporting in primary care centres. Previous studies in the UK CPRD population have found comparable rates of PCOS as we do for our PCOS-phenotype, though our prevalence of PCOS alone is lower, most likely because these studies include PCO alone in their diagnostic criteria for PCOS^70,71^. Cesta et al. also report the mean age at first PCOS diagnosis in Sweden is around 28.0 ± 6.8 years, which is comparable to our own observation (Table 2, 27.6 ± 7.5 years)^31^. In addition, the prevalence of autism in our control female population (0.09%) was lower than that documented in the UK (0.20%)^72^. Again, this may be due to decreased recording of psychiatric conditions by primary care physicians, since these conditions are usually diagnosed by specialists. Another explanation may be that the majority of the women in our population were born before improved diagnostic criteria and screening for autism of the last 20 years, as well as before Asperger’s syndrome was included in the DSM in 1994, which allowed diagnoses to include milder cases of autism (Supplementary Figure 1, Supplementary Table 2). Before the 1980s, the prevalence of autism was 3.3 out of 10,000^73^, and only began to significantly increase in the 1990s. This may also explain the increased age of autism diagnosis in women in our study population, as GPs may now diagnose women who may not have met past diagnostic criteria (Supplementary Figure 1, Supplementary Table 2). Furthermore, girls also tend to be clinically identified later than boys, even with current diagnostic standards^74^. Finally, although none of our controls had a formal PCOS diagnosis, 2.8–3.0% positively qualified for PCOS under NIH or Rotterdam criteria, which also supports the presence of an undiagnosed PCOS population. These false negatives, while potentially attenuating the associations we observe, also increase the likelihood that the statistically significant associations we do find are real.

## Conclusion

This study finds an association between PCOS and autism, which raises the question of whether early treatments or surveillance might be considered for one condition given the presence of the other. Metabolic conditions associated with PCOS can be reversed if caught early, and the social deficits in autism can be reduced with early psychological intervention programmes^75^. Using elevated androgens as well as increased markers of hyperinsulinemia or metabolic disturbances in women with PCOS could, for example, lead to improved screening tools and increased awareness to help identify children with autism earlier. Likewise, assessing risks for metabolic conditions in women with autism may also prevent the development of adverse metabolic outcomes, such as obesity, diabetes, and other complications of PCOS. Of course, the risks of discussing autism associated with PCOS should be carefully weighed to prevent additional stress. We would also underline that our study suggests that autism in children of women with PCOS is still very rare, so the chance of having a child with autism should not be overstated. The association of PCOS and autism therefore may inform new clinical interventions for both disorders.

## Electronic supplementary material

Supplementary Tables

Read Code Lists
