# Supplementary material for: Polycystic ovary syndrome and autism: A test of the prenatal sex steroid theory
Source: Transl Psychiatry. 2018 Aug 1;8:136. doi: 10.1038/s41398-018-0186-7 (PMC6068102; doi:10.1038/s41398-018-0186-7)
Supplement: Supplementary file 1 — Supplementary Tables [file 41398_2018_186_MOESM1_ESM.docx]

**Supplementary Tables and Figures**

| **Supplementary Table 1. Comorbid mental illness and metabolic conditions in women with autism** | | | | | | |
| --- | --- | --- | --- | --- | --- | --- |
|  | ASC | | Controls | | Controls - ASC | |
| *Characteristics* | *Count (n)* | *% Total* | *Count (n)* | *% Total* | *OR (95% CI)* | *P value** |
| **Total** | **971** | --- | **4855** | --- | -- | --- |
| **ASC** |  |  |  |  | -- | -- |
| Autism | 514 | 52.9% | -- | -- | -- | -- |
| Asperger's | 433 | 44.6% | -- | -- | -- | -- |
| PDD-NOS | 24 | 2.5% | -- | -- | -- | -- |
| TOTAL | 971 | 100.0% | -- | -- | -- | -- |
| **Comorbid Mental Illness** |  |  |  |  |  |  |
| Depression | 327 | 33.7% | 681 | 14.0% | **3.11 (2.66 - 3.63)** | < 0.001 |
| Anxiety | 302 | 31.1% | 449 | 11.6% | **4.27 (3.65 - 5.01)** | < 0.001 |
| Schizophrenia | 74 | 7.6% | 23 | 0.5% | **17.08 (10.6 - 27.4)** | < 0.001 |
| **Metabolic Conditions** |  |  |  |  |  |  |
| Obesity | 80 | 8.2% | 110 | 2.3% | **19.5 (12.2 - 31.1)** | < 0.001 |
| DM (Type 1 and 2) | 25 | 2.6% | 36 | 0.7% | **3.54 (2.11 - 5.92)** | < 0.001 |

| **Supplementary Table 2. Age at index ASC diagnosis in women in the CPRD** | | | | | | | | | | |
| --- | --- | --- | --- | --- | --- | --- | --- | --- | --- | --- |
|  | Child Autism | | Autism | | Asperger’s | | PDD-NOS | | Total | |
| *Year of Birth* | *n* | *mean ± s.d* | *n* | *mean ± s.d* | *n* | *mean ± s.d* | *n* | *mean ± s.d* | *n* | *mean ± s.d* |
| **1990 - 1993** | 11 | 12.1 ± 5.4 | 188 | 13.1 ± 5.9 | 161 | 14.2 ± 4.5 | 10 | 15.8 ± 1.5 | 370 | 13.6 ± 5.3 |
| **1980 - 1989** | 4 | 24.0 ± 9.0 | 175 | 17.5 ± 7.8 | 141 | 21.3 ± 5.8 | 7 | 23.6 ± 3.2 | 327 | 19.4 ± 7.2 |
| **1970 - 1979** | 1 | 15 | 70 | 28.2 ± 9.7 | 79 | 33.4 ± 5.8 | 5 | 25 ± 5.3 | 155 | 30.7 ± 8.3 |
| **1960 - 1969** | 0 | --- | 61 | 38.4 ± 13.4 | 52 | 43.9 ± 5.0 | 2 | 44.5 ± 3.5 | 115 | 41 ± 10.7 |
| Total | 16 | 15.3 ± 7.9 | 510 | 19.8 ± 11.8 | 433 | 23.6 ± 11.4 | 24 | 22.4± 8.5 | 967* | 21.5 ± 11.7 |

* n = 971 total women in study, n = 4 missing values for age at index diagnosis

**Supplementary Figure 1. Mean age at ASC diagnosis in women in the CPRD**

Mean age at first documented CPRD diagnosis of ASC, separated according to historic diagnostic categories: Child Autism, Autism, Asperger’s, PDD-NOS (pervasive developmental disorder, not otherwise specified). Error bars show s.d. of the mean. See supplementary table 2 for n of each group.

| **Supplementary Table 3. Characteristics of women with PCOS and PCOS phenotype** | | | | | | |
| --- | --- | --- | --- | --- | --- | --- |
|  | PCOS | |  | PCOS Phenotype | |  |
| *Characteristics* | *Count (n)* | *% Total* | *Mean age ± s.d* | *Count (n)* | *% Total* | *Mean age ± s.d* |
| **Total** | 10 201 | --- | 34.1 ± 8.2 | 26 263 | --- | 35.5 ± 8.5*** |
| **ASC** | 29 | 0.28% | 13.32 ± 7.8 | 45** | 0.17%** | 16.2 ± 8.9 |
| **Other Psychiatric Conditions** |  |  |  |  |  |  |
| Depression | 3 302 | 32.4% | 26.7 ± 7.7 | 8 700 | 33.1% | 27.1 ± 7.6* |
| Anxiety | 2 158 | 21.2% | 26.7 ± 7.8 | 5 608 | 21.4% | 27.2 ± 8.2* |
| Schizophrenia | 90 | 0.88% | 28.4 ± 8.7 | 233 | 0.88% | 27.8 ± 8.2 |
| **PCOS** |  |  |  |  |  |  |
| PCOS Read Code | 10 201 | 100.0% | 27.6 ± 7.5 | 10 201 | 38.8% | 27.6 ± 7.5 |
| NIH | 4 126 | 40.4% | --- | 14 565 | 55.5%*** | --- |
| Rotterdam | 4 834 | 47.4% | --- | 19 891 | 75.7%*** | --- |
| Menstrual Irregularities | 5 155 | 50.5% | 24.7 ± 6.7 | 15 238 | 58.0%*** | 25.4 ± 6.6*** |
| Hyperandrogenemia | 5 954 | 58.4% | 23.7 ± 8.0 | 16 393 | 62.4%*** | 24.6 ± 8.0*** |
| PCO | 3 582 | 35.1% | 26.3 ± 6.7 | 19 644 | 74.8%*** | 26.7 ± 6.5 |
| **Metabolic Conditions** |  |  |  |  |  |  |
| Diabetes | 363 | 3.6% | 33.7 ± 10.0 | 898 | 3.4% | 33.8 ± 10.9 |
| Obesity | 2 215 | 21.7% | 27.1 ± 8.6 | 4 904 | 18.7%*** | 27.6 ± 8.5* |

Student’s two-tailed t-test and Chi-square test p-values PCOS vs PCOS Phenotype: * p-value < 0.05, ** p-value < 0.01, *** p-value < 0.001

| **Supplementary Table 4. PCOS characteristics in women with PCOS and controls in the CPRD** | | | | | |
| --- | --- | --- | --- | --- | --- |
|  | PCOS Phenotype^1^ | | Controls | | Controls - PCOS Phenotype |
| *Characteristics* | *Count (n)* | *% Total* | *Count (n)* | *% Total* | *Unadjusted OR (95% CI)* |
| **Total** | 26 263 |  | 130 717 |  | --- |
| **PCOS** |  |  |  |  |  |
| *PCOS Read Code* | 10 201 | 38.8% | 0 | 0.0% |  |
| *NIH* | 14 565 | 55.5% | 3 648 | 2.8% | --- |
| *Rotterdam* | 19 891 | 75.7% | 3 877 | 3.0% | --- |
| *Menstrual Irregularities* | 15 238 | 58.0% | 12 603 | 9.6% | **13.0 (12.6 – 13.4)***** |
| *Hyperandrogenemia* | 16 393 | 62.4% | 17 823 | 13.6% | **16.2 (15.7 – 16.7)***** |
| *PCO* | 19 644 | 74.8% | 1 615 | 1.2% | **237.2 (224.2 – 251.0)***** |

* p-value < 0.05, ** p-value < 0.01, *** p-value < 0.001

| **Supplementary Table 5. Characteristics of PCOS mothers and PCOS Phenotype mothers** | | | | |
| --- | --- | --- | --- | --- |
|  | PCOS | | PCOS Phenotype | |
| *Maternal characteristics* | *Count* | *% Total* | *Count* | *% Total* |
| **Total** | **3 023** | --- | **8 588** | --- |
| **Avg. maternal age** | 36.5 ± 6.7 | --- | 37.8 ± 7.2*** | --- |
| **Metabolic conditions (Lifetime)** |  |  |  |  |
| *Diabetes Mellitus Type 1 and 2* | 113 | 3.7% | 298 | 3.5% |
| *Obesity* | 682 | 22.5% | 1 686*** | 19.6% |
| **Pregnancy complications** |  |  |  |  |
| *Childbirth complications* | 36 | 1.2% | 106 | 1.2% |
| *Gestational diabetes* | 112 | 3.7% | 226*** | 2.6% |
| *Infertility diagnosis* | 704 | 23.2% | 2 062 | 24.0% |
| *Pre-eclampsia for this pregnancy* | 0 | 0% | 1 | 0.05% |
| **Psychiatric conditions (Lifetime)** |  |  |  |  |
| *Depression* | 1 218 | 40.2% | 3 528 | 41.1% |
| *Anxiety Disorders* | 729 | 24.1% | 2 058 | 24.0% |
| *Schizophrenia and mental illness* | 19 | 0.6% | 54 | 0.6% |
| *ASC* | 1 | 0% | 4 | 0.05% |

Student’s two-tailed t-test and Chi-square test p-values PCOS vs PCOS Phenotype: * p-value < 0.05, ** p-value < 0.01, *** p-value < 0.001
