# Supplementary material for: Polycystic ovary syndrome and autism: A test of the prenatal sex steroid theory
Source: Transl Psychiatry. 2018 Aug 1;8:136. doi: 10.1038/s41398-018-0186-7 (PMC6068102; doi:10.1038/s41398-018-0186-7)
Supplement: Supplementary file 2 — Read Code Lists [file 41398_2018_186_MOESM2_ESM.docx]

**Appendix A:**

*Read Codes*

*EXPOSURE AND OUTCOME*

**Polycystic Ovary Syndrome (PCOS):**

| Read Code | Description |
| --- | --- |
| C164.12 | Stein-Leventhal syndrome |
| C165.00 | Polycystic ovarian syndrome |

**OR**

| Read Code | Description |
| --- | --- |
| C164.00 | Polycystic ovaries |

**AND**

Hyperandrogenism:

| Read Code | Description |
| --- | --- |
| C161000 | Hypersecretion of ovarian androgen |
| Myu6300 | [X]Other androgenic alopecia |
| M241.00 | Hirsutism -- hypertrichosis |
| M261z00 | Other acne NOS |
| M261z00 | Acne, unspecified |
| Myo6F00 | [X]Acne, unspecified |
| M261000 | Acne vulgaris |

Blood Test Events:

| Read Code | Description |
| --- | --- |
| 4Q2F.00 | Calculated free testosterone |
| 4473.00 | Serum testosterone |
| 4Q2E.00 | Free testosterone level |
| 4474.00 | Free androgen index |
| 4474100 | Free androgen index abnormal |
| 4473100 | Serum testosterone level abnormal |

AND/OR

Menstrual cycle disturbance:

| Read Code | Description |
| --- | --- |
| K594.00 | Irregular menstrual cycle |
| K591300 | Secondary oligomenorrhoea |
| K591200 | Primary oligomenorrhoea |
| K591100 | Oligomenorrhoea |
| K590.11 | Amenorrhoea |
| K590z00 | Amenorrhoea NOS |
| Kyu9C00 | [X]Other specified irregular menstruation |
| K591z00 | Scanty or infrequent menstruation NOS |
| K591.11 | Infrequent menstruation |
| K591.00 | Scanty or infrequent menstruation |
| K590.00 | Absence of menstruation |

Exclusion criteria:

| Read Code | Description |
| --- | --- |
| C150.00 | Cushing's syndrome |
| C150111 | Drug-induced Cushing's syndrome |
| C150200 | Pituitary dependent Cushing's syndrome |
| C150z00 | Cushing's syndrome NOS |
| C150300 | Ectopic ACTH secretion causing Cushing's syndrome |
| C150000 | Idiopathic Cushing's syndrome |
| Cyo4500 | [X] Other Cushing's syndrome |
| C152812 | Congenital adrenal hyperplasia |
| C150400 | Nelson's syndrome |
| C152.00 | Adrenogenital disorders |
| C152812 | Congenital adrenal hyperplasia NEC |

**Autism Spectrum Conditions (ASC):**

| Read Code | Description |
| --- | --- |
| E140.12 | Autism |
| Eu84011 | [X] Autistic disorder |
| E140.00 | Infantile autism |
| Eu84z11 | [X] Autistic spectrum disorder |
| Eu84000 | [X] Childhood autism |
| E140.13 | Chidlhood autism |
| E140000 | Active infantile autism |
| E140.11 | Kanner's syndrome |
| E140100 | Residual infantile autism |
| E140z00 | Infantile autism NOS |
| E141.00 | Disintegrative psychosis |
| E141.11 | Heller's Syndrome |
| E141z00 | Residual disintegrative psychoses |
| E84012 | [X] Infantile autism |
| Eu84013 | [X] Infantile psychosis |
| Eu84112 | [X] Mental retardation with autistic features |
| Eu84300 | [X] Other childhood disintegrative disorder |
| Eu84311 | [X] Dementia infantalis |
| Eu84312 | [X] Disintegrative psychosis |
| Eu84313 | [X] Heller's syndrome |
| Eu84500 | [X] Asperger's syndrome |
| Eu84.00 | [X] Pervasive developmental disorders |
| Eu84100 | [X] Atypical autism |
| Eu84y00 | [X] Pervasive developmental disorder, unspecified |

*COVARIATES*

**Obesity:**

| Read code | Description |
| --- | --- |
| 222A.00 | O/E - obese |
| 22A5.11 | O/E - obese |
| 22K5.00 | Body mass index 30+ - obesity |
| 22K7.00 | Body mass index 40+ - severely obese |
| 66C..00 | Obesity monitoring |
| 66C4.00 | Has seen dietician - obesity |
| 66C6.00 | Treatment of obesity started |
| 66CM.00 | Risk health associ overweight and obesity, at increased risk |
| 9OK3.00 | Obesity monitoring default |
| C38..00 | Obesity and other hyperalimentation |
| C380.00 | Obesity |
| C380000 | Obesity due to excess calories |
| C380200 | Extreme obesity with alveolar hypoventilation |
| C380300 | Morbid obesity |
| C380400 | Central obesity |
| C380500 | Generalised obesity |
| C380700 | Lifelong obesity |
| C38y011 | Obesity hypoventilation syndrome |
| C38z.00 | Obesity and other hyperalimentation NOS |
| C38z000 | Simple obesity NOS |
| Cyu7.00 | [X]Obesity and other hyperalimentation |
| Cyu7000 | [X]Other obesity |
| ZC2CM00 | Dietary advice for obesity |

**Infertility**

| Read code | Description |
| --- | --- |
| 8C8Z.00 | Treatment for infertility NOS |
| K5B5100 | Secondary vaginal infertility |
| K5B3100 | Secondary uterine infertility |
| K5B2100 | Secondary tubal infertility |
| K5B1100 | Secondary pituitary - hypothalamic infertility |
| K5By100 | Secondary infertility unspecified |
| K5B4100 | Secondary cervical infertility |
| K5B0100 | Secondary anovulatory infertility |
| K5B3000 | Primary uterine infertility |
| K5B2000 | Primary tubal infertility |
| K5B1000 | Primary pituitary - hypothalamic infertility |
| K5By000 | Primary infertility unspecified |
| K5B4000 | Primary cervical infertility |
| K5B0000 | Primary anovulatory infertility |
| K5Byz00 | Other female infertility NOS |
| K5By.00 | Other female infertility |
| 1AZ2.11 | Infertility problem |
| K5B..00 | Infertility - female |
| 8C82.00 | Female infertility therapy |
| 3189100 | Female infertility test abnormal |
| K5B5.00 | Female infertility of vaginal origin |
| K5B3z00 | Female infertility of uterine origin NOS |
| K5B3.00 | Female infertility of uterine origin |
| K5B2z00 | Female infertility of tubal origin NOS |
| K5B2.00 | Female infertility of tubal origin |
| K5B1.00 | Female infertility of pituitary - hypothalamic origin |
| K5B1z00 | Female infertility of pituitary - hypothalamic cause NOS |
| K5B4.00 | Female infertility of cervical origin |
| K5B0z00 | Female infertility of anovulatory origin NOS |
| K5B0.00 | Female infertility of anovulatory origin |
| K5Bz.00 | Female infertility NOS |
| K5B7.00 | Female infertility due to diminished ovarian reserve |
| Kyu9G00 | [X]Female infertility of other origin |
| ZV26z00 | [V]Unspecified infertility management |
| ZV23000 | [V]Pregnancy with history of infertility |
| ZV26y00 | [V]Other specified infertility management |
| ZV26.00 | [V]Infertility management |

**Gestational diabetes**

| Read code | Description |
| --- | --- |
| L180.00 | Diabetes mellitus during pregnancy/childbirth/puerperium |
| L180100 | Diabetes mellitus during pregnancy - baby delivered |
| L180300 | Diabetes mellitus during pregnancy - baby not yet delivered |
| L180800 | Diabetes mellitus arising in pregnancy |
| L180811 | Gestational diabetes mellitus |
| L180900 | Gestational diabetes mellitus |
| L180z00 | Diabetes mellitus in pregnancy/childbirth/puerperium NOS |

**Pre-eclampsia**

| Read code | Description |
| --- | --- |
| L124000 | Mild or unspecified pre-eclampsia unspecified |
| L124100 | Mild or unspecified pre-eclampsia - delivered |
| L124200 | Mild or unspecified pre-eclampsia - delivered with p/n comp |
| L124300 | Mild or unspecified pre-eclampsia - not delivered |
| L124400 | Mild or unspecified pre-eclampsia with p/n complication |
| L124z00 | Mild or unspecified pre-eclampsia NOS |
| L125000 | Severe pre-eclampsia unspecified |
| L125100 | Severe pre-eclampsia - delivered |
| L125200 | Severe pre-eclampsia - delivered with postnatal complication |
| L125300 | Severe pre-eclampsia - not delivered |
| L125400 | Severe pre-eclampsia with postnatal complication |
| L125z00 | Severe pre-eclampsia NOS |

**Type I and II Diabetes**

| Read code | Description |
| --- | --- |
| 1434 | H/O: diabetes mellitus |
| 13L4.11 | Diabetic child |
| 66A3.00 | Diabetic on diet only |
| 66A4.00 | Diabetic on oral treatment |
| 66A5.00 | Diabetic on insulin |
| 66AI.00 | Diabetic - good control |
| 66AJ.00 | Diabetic - poor control |
| 66AJ.11 | Unstable diabetes |
| 66AJ100 | Brittle diabetes |
| 66AJz00 | Diabetic - poor control NOS |
| 66AK.00 | Diabetic - cooperative patient |
| 66AL.00 | Diabetic-uncooperative patient |
| 66As.00 | Diabetic on subcutaneous treatment |
| 66AV.00 | Diabetic on insulin and oral treatment |
| C10..00 | Diabetes mellitus |
| C100.00 | Diabetes mellitus with no mention of complication |
| C100000 | Diabetes mellitus, juvenile type, no mention of complication |
| C100011 | Insulin dependent diabetes mellitus |
| C100100 | Diabetes mellitus, adult onset, no mention of complication |
| C100111 | Maturity onset diabetes |
| C100112 | Non-insulin dependent diabetes mellitus |
| C100z00 | Diabetes mellitus NOS with no mention of complication |
| C101.00 | Diabetes mellitus with ketoacidosis |
| C101000 | Diabetes mellitus, juvenile type, with ketoacidosis |
| C101100 | Diabetes mellitus, adult onset, with ketoacidosis |
| C101y00 | Other specified diabetes mellitus with ketoacidosis |
| C101z00 | Diabetes mellitus NOS with ketoacidosis |
| C102.00 | Diabetes mellitus with hyperosmolar coma |
| C102000 | Diabetes mellitus, juvenile type, with hyperosmolar coma |
| C102100 | Diabetes mellitus, adult onset, with hyperosmolar coma |
| C102z00 | Diabetes mellitus NOS with hyperosmolar coma |
| C103.00 | Diabetes mellitus with ketoacidotic coma |
| C103000 | Diabetes mellitus, juvenile type, with ketoacidotic coma |
| C103100 | Diabetes mellitus, adult onset, with ketoacidotic coma |
| C103y00 | Other specified diabetes mellitus with coma |
| C103z00 | Diabetes mellitus NOS with ketoacidotic coma |
| C104.00 | Diabetes mellitus with renal manifestation |
| C104000 | Diabetes mellitus, juvenile type, with renal manifestation |
| C104100 | Diabetes mellitus, adult onset, with renal manifestation |
| C104y00 | Other specified diabetes mellitus with renal complications |
| C104z00 | Diabetes mellitus with nephropathy NOS |
| C105.00 | Diabetes mellitus with ophthalmic manifestation |
| C105000 | Diabetes mellitus, juvenile type, + ophthalmic manifestation |
| C105100 | Diabetes mellitus, adult onset, + ophthalmic manifestation |
| C105y00 | Other specified diabetes mellitus with ophthalmic complicatn |
| C105z00 | Diabetes mellitus NOS with ophthalmic manifestation |
| C106.00 | Diabetes mellitus with neurological manifestation |
| C106.12 | Diabetes mellitus with neuropathy |
| C106.13 | Diabetes mellitus with polyneuropathy |
| C106000 | Diabetes mellitus, juvenile, + neurological manifestation |
| C106100 | Diabetes mellitus, adult onset, + neurological manifestation |
| C106y00 | Other specified diabetes mellitus with neurological comps |
| C106z00 | Diabetes mellitus NOS with neurological manifestation |
| C107.00 | Diabetes mellitus with peripheral circulatory disorder |
| C107.11 | Diabetes mellitus with gangrene |
| C107.12 | Diabetes with gangrene |
| C107000 | Diabetes mellitus, juvenile +peripheral circulatory disorder |
| C107100 | Diabetes mellitus, adult, + peripheral circulatory disorder |
| C107200 | Diabetes mellitus, adult with gangrene |
| C107300 | IDDM with peripheral circulatory disorder |
| C107400 | NIDDM with peripheral circulatory disorder |
| C107z00 | Diabetes mellitus NOS with peripheral circulatory disorder |
| C108.00 | Insulin dependent diabetes mellitus |
| C108.11 | IDDM-Insulin dependent diabetes mellitus |
| C108.12 | Type 1 diabetes mellitus |
| C108.13 | Type I diabetes mellitus |
| C108000 | Insulin-dependent diabetes mellitus with renal complications |
| C108011 | Type I diabetes mellitus with renal complications |
| C108012 | Type 1 diabetes mellitus with renal complications |
| C108100 | Insulin-dependent diabetes mellitus with ophthalmic comps |
| C108112 | Type 1 diabetes mellitus with ophthalmic complications |
| C108200 | Insulin-dependent diabetes mellitus with neurological comps |
| C108211 | Type I diabetes mellitus with neurological complications |
| C108212 | Type 1 diabetes mellitus with neurological complications |
| C108300 | Insulin dependent diabetes mellitus with multiple complicatn |
| C108311 | Type I diabetes mellitus with multiple complications |
| C108400 | Unstable insulin dependent diabetes mellitus |
| C108411 | Unstable type I diabetes mellitus |
| C108412 | Unstable type 1 diabetes mellitus |
| C108500 | Insulin dependent diabetes mellitus with ulcer |
| C108511 | Type I diabetes mellitus with ulcer |
| C108512 | Type 1 diabetes mellitus with ulcer |
| C108600 | Insulin dependent diabetes mellitus with gangrene |
| C108700 | Insulin dependent diabetes mellitus with retinopathy |
| C108711 | Type I diabetes mellitus with retinopathy |
| C108712 | Type 1 diabetes mellitus with retinopathy |
| C108800 | Insulin dependent diabetes mellitus - poor control |
| C108811 | Type I diabetes mellitus - poor control |
| C108812 | Type 1 diabetes mellitus - poor control |
| C108900 | Insulin dependent diabetes maturity onset |
| C108911 | Type I diabetes mellitus maturity onset |
| C108912 | Type 1 diabetes mellitus maturity onset |
| C108A00 | Insulin-dependent diabetes without complication |
| C108A11 | Type I diabetes mellitus without complication |
| C108B00 | Insulin dependent diabetes mellitus with mononeuropathy |
| C108B11 | Type I diabetes mellitus with mononeuropathy |
| C108C00 | Insulin dependent diabetes mellitus with polyneuropathy |
| C108D00 | Insulin dependent diabetes mellitus with nephropathy |
| C108D11 | Type I diabetes mellitus with nephropathy |
| C108E00 | Insulin dependent diabetes mellitus with hypoglycaemic coma |
| C108E11 | Type I diabetes mellitus with hypoglycaemic coma |
| C108E12 | Type 1 diabetes mellitus with hypoglycaemic coma |
| C108F00 | Insulin dependent diabetes mellitus with diabetic cataract |
| C108F11 | Type I diabetes mellitus with diabetic cataract |
| C108H00 | Insulin dependent diabetes mellitus with arthropathy |
| C108H11 | Type I diabetes mellitus with arthropathy |
| C108J11 | Type I diabetes mellitus with neuropathic arthropathy |
| C108J12 | Type 1 diabetes mellitus with neuropathic arthropathy |
| C108y00 | Other specified diabetes mellitus with multiple comps |
| C108z00 | Unspecified diabetes mellitus with multiple complications |
| C109.00 | Non-insulin dependent diabetes mellitus |
| C109.11 | NIDDM - Non-insulin dependent diabetes mellitus |
| C109.12 | Type 2 diabetes mellitus |
| C109.13 | Type II diabetes mellitus |
| C109000 | Non-insulin-dependent diabetes mellitus with renal comps |
| C109011 | Type II diabetes mellitus with renal complications |
| C109012 | Type 2 diabetes mellitus with renal complications |
| C109100 | Non-insulin-dependent diabetes mellitus with ophthalm comps |
| C109111 | Type II diabetes mellitus with ophthalmic complications |
| C109112 | Type 2 diabetes mellitus with ophthalmic complications |
| C109200 | Non-insulin-dependent diabetes mellitus with neuro comps |
| C109211 | Type II diabetes mellitus with neurological complications |
| C109212 | Type 2 diabetes mellitus with neurological complications |
| C109300 | Non-insulin-dependent diabetes mellitus with multiple comps |
| C109312 | Type 2 diabetes mellitus with multiple complications |
| C109400 | Non-insulin dependent diabetes mellitus with ulcer |
| C109411 | Type II diabetes mellitus with ulcer |
| C109412 | Type 2 diabetes mellitus with ulcer |
| C109500 | Non-insulin dependent diabetes mellitus with gangrene |
| C109511 | Type II diabetes mellitus with gangrene |
| C109512 | Type 2 diabetes mellitus with gangrene |
| C109600 | Non-insulin-dependent diabetes mellitus with retinopathy |
| C109611 | Type II diabetes mellitus with retinopathy |
| C109612 | Type 2 diabetes mellitus with retinopathy |
| C109700 | Non-insulin dependent diabetes mellitus - poor control |
| C109711 | Type II diabetes mellitus - poor control |
| C109712 | Type 2 diabetes mellitus - poor control |
| C109900 | Non-insulin-dependent diabetes mellitus without complication |
| C109911 | Type II diabetes mellitus without complication |
| C109912 | Type 2 diabetes mellitus without complication |
| C109A00 | Non-insulin dependent diabetes mellitus with mononeuropathy |
| C109A11 | Type II diabetes mellitus with mononeuropathy |
| C109B00 | Non-insulin dependent diabetes mellitus with polyneuropathy |
| C109B11 | Type II diabetes mellitus with polyneuropathy |
| C109C00 | Non-insulin dependent diabetes mellitus with nephropathy |
| C109C11 | Type II diabetes mellitus with nephropathy |
| C109C12 | Type 2 diabetes mellitus with nephropathy |
| C109D00 | Non-insulin dependent diabetes mellitus with hypoglyca coma |
| C109D11 | Type II diabetes mellitus with hypoglycaemic coma |
| C109D12 | Type 2 diabetes mellitus with hypoglycaemic coma |
| C109E00 | Non-insulin depend diabetes mellitus with diabetic cataract |
| C109E11 | Type II diabetes mellitus with diabetic cataract |
| C109E12 | Type 2 diabetes mellitus with diabetic cataract |
| C109F11 | Type II diabetes mellitus with peripheral angiopathy |
| C109F12 | Type 2 diabetes mellitus with peripheral angiopathy |
| C109G00 | Non-insulin dependent diabetes mellitus with arthropathy |
| C109G11 | Type II diabetes mellitus with arthropathy |
| C109G12 | Type 2 diabetes mellitus with arthropathy |
| C109H11 | Type II diabetes mellitus with neuropathic arthropathy |
| C109H12 | Type 2 diabetes mellitus with neuropathic arthropathy |
| C109J00 | Insulin treated Type 2 diabetes mellitus |
| C109J11 | Insulin treated non-insulin dependent diabetes mellitus |
| C109J12 | Insulin treated Type II diabetes mellitus |
| C10A.00 | Malnutrition-related diabetes mellitus |
| C10A000 | Malnutrition-related diabetes mellitus with coma |
| C10A500 | Malnutritn-relat diabetes melitus wth periph circul complctn |
| C10C.00 | Diabetes mellitus autosomal dominant |
| C10C.11 | Maturity onset diabetes in youth |
| C10C.12 | Maturity onset diabetes in youth type 1 |
| C10D.00 | Diabetes mellitus autosomal dominant type 2 |
| C10D.11 | Maturity onset diabetes in youth type 2 |
| C10E.00 | Type 1 diabetes mellitus |
| C10E.11 | Type I diabetes mellitus |
| C10E.12 | Insulin dependent diabetes mellitus |
| C10E000 | Type 1 diabetes mellitus with renal complications |
| C10E012 | Insulin-dependent diabetes mellitus with renal complications |
| C10E100 | Type 1 diabetes mellitus with ophthalmic complications |
| C10E111 | Type I diabetes mellitus with ophthalmic complications |
| C10E112 | Insulin-dependent diabetes mellitus with ophthalmic comps |
| C10E200 | Type 1 diabetes mellitus with neurological complications |
| C10E212 | Insulin-dependent diabetes mellitus with neurological comps |
| C10E300 | Type 1 diabetes mellitus with multiple complications |
| C10E311 | Type I diabetes mellitus with multiple complications |
| C10E312 | Insulin dependent diabetes mellitus with multiple complicat |
| C10E400 | Unstable type 1 diabetes mellitus |
| C10E411 | Unstable type I diabetes mellitus |
| C10E412 | Unstable insulin dependent diabetes mellitus |
| C10E500 | Type 1 diabetes mellitus with ulcer |
| C10E511 | Type I diabetes mellitus with ulcer |
| C10E512 | Insulin dependent diabetes mellitus with ulcer |
| C10E600 | Type 1 diabetes mellitus with gangrene |
| C10E611 | Type I diabetes mellitus with gangrene |
| C10E612 | Insulin dependent diabetes mellitus with gangrene |
| C10E700 | Type 1 diabetes mellitus with retinopathy |
| C10E711 | Type I diabetes mellitus with retinopathy |
| C10E712 | Insulin dependent diabetes mellitus with retinopathy |
| C10E800 | Type 1 diabetes mellitus - poor control |
| C10E811 | Type I diabetes mellitus - poor control |
| C10E812 | Insulin dependent diabetes mellitus - poor control |
| C10E900 | Type 1 diabetes mellitus maturity onset |
| C10E911 | Type I diabetes mellitus maturity onset |
| C10E912 | Insulin dependent diabetes maturity onset |
| C10EA00 | Type 1 diabetes mellitus without complication |
| C10EA11 | Type I diabetes mellitus without complication |
| C10EA12 | Insulin-dependent diabetes without complication |
| C10EB00 | Type 1 diabetes mellitus with mononeuropathy |
| C10EC00 | Type 1 diabetes mellitus with polyneuropathy |
| C10EC11 | Type I diabetes mellitus with polyneuropathy |
| C10EC12 | Insulin dependent diabetes mellitus with polyneuropathy |
| C10ED00 | Type 1 diabetes mellitus with nephropathy |
| C10ED12 | Insulin dependent diabetes mellitus with nephropathy |
| C10EE00 | Type 1 diabetes mellitus with hypoglycaemic coma |
| C10EE12 | Insulin dependent diabetes mellitus with hypoglycaemic coma |
| C10EF00 | Type 1 diabetes mellitus with diabetic cataract |
| C10EF12 | Insulin dependent diabetes mellitus with diabetic cataract |
| C10EG00 | Type 1 diabetes mellitus with peripheral angiopathy |
| C10EH00 | Type 1 diabetes mellitus with arthropathy |
| C10EJ00 | Type 1 diabetes mellitus with neuropathic arthropathy |
| C10EL00 | Type 1 diabetes mellitus with persistent microalbuminuria |
| C10EL11 | Type I diabetes mellitus with persistent microalbuminuria |
| C10EM00 | Type 1 diabetes mellitus with ketoacidosis |
| C10EM11 | Type I diabetes mellitus with ketoacidosis |
| C10EN00 | Type 1 diabetes mellitus with ketoacidotic coma |
| C10EN11 | Type I diabetes mellitus with ketoacidotic coma |
| C10EP00 | Type 1 diabetes mellitus with exudative maculopathy |
| C10EP11 | Type I diabetes mellitus with exudative maculopathy |
| C10EQ00 | Type 1 diabetes mellitus with gastroparesis |
| C10EQ11 | Type I diabetes mellitus with gastroparesis |
| C10ER00 | Latent autoimmune diabetes mellitus in adult |
| C10F.00 | Type 2 diabetes mellitus |
| C10F.11 | Type II diabetes mellitus |
| C10F000 | Type 2 diabetes mellitus with renal complications |
| C10F011 | Type II diabetes mellitus with renal complications |
| C10F100 | Type 2 diabetes mellitus with ophthalmic complications |
| C10F111 | Type II diabetes mellitus with ophthalmic complications |
| C10F200 | Type 2 diabetes mellitus with neurological complications |
| C10F211 | Type II diabetes mellitus with neurological complications |
| C10F300 | Type 2 diabetes mellitus with multiple complications |
| C10F311 | Type II diabetes mellitus with multiple complications |
| C10F400 | Type 2 diabetes mellitus with ulcer |
| C10F411 | Type II diabetes mellitus with ulcer |
| C10F500 | Type 2 diabetes mellitus with gangrene |
| C10F511 | Type II diabetes mellitus with gangrene |
| C10F600 | Type 2 diabetes mellitus with retinopathy |
| C10F611 | Type II diabetes mellitus with retinopathy |
| C10F700 | Type 2 diabetes mellitus - poor control |
| C10F711 | Type II diabetes mellitus - poor control |
| C10F900 | Type 2 diabetes mellitus without complication |
| C10F911 | Type II diabetes mellitus without complication |
| C10FA00 | Type 2 diabetes mellitus with mononeuropathy |
| C10FA11 | Type II diabetes mellitus with mononeuropathy |
| C10FB00 | Type 2 diabetes mellitus with polyneuropathy |
| C10FB11 | Type II diabetes mellitus with polyneuropathy |
| C10FC00 | Type 2 diabetes mellitus with nephropathy |
| C10FC11 | Type II diabetes mellitus with nephropathy |
| C10FD00 | Type 2 diabetes mellitus with hypoglycaemic coma |
| C10FD11 | Type II diabetes mellitus with hypoglycaemic coma |
| C10FE00 | Type 2 diabetes mellitus with diabetic cataract |
| C10FE11 | Type II diabetes mellitus with diabetic cataract |
| C10FF00 | Type 2 diabetes mellitus with peripheral angiopathy |
| C10FF11 | Type II diabetes mellitus with peripheral angiopathy |
| C10FG00 | Type 2 diabetes mellitus with arthropathy |
| C10FG11 | Type II diabetes mellitus with arthropathy |
| C10FH00 | Type 2 diabetes mellitus with neuropathic arthropathy |
| C10FH11 | Type II diabetes mellitus with neuropathic arthropathy |
| C10FJ00 | Insulin treated Type 2 diabetes mellitus |
| C10FJ11 | Insulin treated Type II diabetes mellitus |
| C10FL00 | Type 2 diabetes mellitus with persistent proteinuria |
| C10FL11 | Type II diabetes mellitus with persistent proteinuria |
| C10FM00 | Type 2 diabetes mellitus with persistent microalbuminuria |
| C10FM11 | Type II diabetes mellitus with persistent microalbuminuria |
| C10FN00 | Type 2 diabetes mellitus with ketoacidosis |
| C10FN11 | Type II diabetes mellitus with ketoacidosis |
| C10FP00 | Type 2 diabetes mellitus with ketoacidotic coma |
| C10FP11 | Type II diabetes mellitus with ketoacidotic coma |
| C10FQ00 | Type 2 diabetes mellitus with exudative maculopathy |
| C10FR00 | Type 2 diabetes mellitus with gastroparesis |
| C10FS00 | Maternally inherited diabetes mellitus |
| C10G.00 | Secondary pancreatic diabetes mellitus |
| C10G000 | Secondary pancreatic diabetes mellitus without complication |
| C10M.00 | Lipoatrophic diabetes mellitus |
| C10y.00 | Diabetes mellitus with other specified manifestation |
| C10y100 | Diabetes mellitus, adult, + other specified manifestation |
| C10yy00 | Other specified diabetes mellitus with other spec comps |
| C10yz00 | Diabetes mellitus NOS with other specified manifestation |
| C10z.00 | Diabetes mellitus with unspecified complication |
| C10z000 | Diabetes mellitus, juvenile type, + unspecified complication |
| C10z100 | Diabetes mellitus, adult onset, + unspecified complication |
| C10zy00 | Other specified diabetes mellitus with unspecified comps |
| C10zz00 | Diabetes mellitus NOS with unspecified complication |
| Cyu2.00 | [X]Diabetes mellitus |
| Cyu2000 | [X]Other specified diabetes mellitus |
| Cyu2300 | [X]Unspecified diabetes mellitus with renal complications |
| L180500 | Pre-existing diabetes mellitus, insulin-dependent |
| L180600 | Pre-existing diabetes mellitus, non-insulin-dependent |
| L180X00 | Pre-existing diabetes mellitus, unspecified |

**Depression**

| Read code | Description |
| --- | --- |
| 6659000 | Antidepressant drug treatment started |
| 1B17.00 | Depressed |
| 1B1U.00 | Symptoms of depression |
| 1B1U.11 | Depressive symptoms |
| 1BT..00 | Depressed mood |
| 62T1.00 | Puerperal depression |
| 665A000 | Antidepressant drug treatment stopped |
| E11..12 | Depressive psychoses |
| E112.00 | Single major depressive episode |
| E112.11 | Agitated depression |
| E112.12 | Endogenous depression first episode |
| E112.13 | Endogenous depression first episode |
| E112.14 | Endogenous depression |
| E112000 | Single major depressive episode, unspecified |
| E112100 | Single major depressive episode, mild |
| E112200 | Single major depressive episode, moderate |
| E112300 | Single major depressive episode, severe, without psychosis |
| E112400 | Single major depressive episode, severe, with psychosis |
| E112500 | Single major depressive episode, partial or unspec remission |
| E112600 | Single major depressive episode, in full remission |
| E112z00 | Single major depressive episode NOS |
| E113.00 | Recurrent major depressive episode |
| E113.11 | Endogenous depression - recurrent |
| E113000 | Recurrent major depressive episodes, unspecified |
| E113100 | Recurrent major depressive episodes, mild |
| E113200 | Recurrent major depressive episodes, moderate |
| E113300 | Recurrent major depressive episodes, severe, no psychosis |
| E113400 | Recurrent major depressive episodes, severe, with psychosis |
| E113500 | Recurrent major depressive episodes,partial/unspec remission |
| E113600 | Recurrent major depressive episodes, in full remission |
| E113700 | Recurrent depression |
| E113z00 | Recurrent major depressive episode NOS |
| E114.11 | Manic-depressive - now manic |
| E115.00 | Bipolar affective disorder, currently depressed |
| E115.11 | Manic-depressive - now depressed |
| E115000 | Bipolar affective disorder, currently depressed, unspecified |
| E115100 | Bipolar affective disorder, currently depressed, mild |
| E115200 | Bipolar affective disorder, currently depressed, moderate |
| E115300 | Bipolar affect disord, now depressed, severe, no psychosis |
| E115400 | Bipolar affect disord, now depressed, severe with psychosis |
| E115500 | Bipolar affect disord, now depressed, part/unspec remission |
| E115600 | Bipolar affective disorder, now depressed, in full remission |
| E115z00 | Bipolar affective disorder, currently depressed, NOS |
| E11y.00 | Other and unspecified manic-depressive psychoses |
| E11y000 | Unspecified manic-depressive psychoses |
| E11y200 | Atypical depressive disorder |
| E11y300 | Other mixed manic-depressive psychoses |
| E11yz00 | Other and unspecified manic-depressive psychoses NOS |
| E11z200 | Masked depression |
| E135.00 | Agitated depression |
| E200300 | Anxiety with depression |
| E204.00 | Neurotic depression reactive type |
| E204.11 | Postnatal depression |
| E211200 | Depressive personality disorder |
| E2B..00 | Depressive disorder NEC |
| E2B1.00 | Chronic depression |
| Eu31.11 | [X]Manic-depressive illness |
| Eu31.12 | [X]Manic-depressive psychosis |
| Eu31300 | [X]Bipolar affect disorder cur epi mild or moderate depressn |
| Eu31400 | [X]Bipol aff disord, curr epis sev depress, no psychot symp |
| Eu32.00 | [X]Depressive episode |
| Eu32000 | [X]Mild depressive episode |
| Eu32100 | [X]Moderate depressive episode |
| Eu32200 | [X]Severe depressive episode without psychotic symptoms |
| Eu32211 | [X]Single episode agitated depressn w'out psychotic symptoms |
| Eu32212 | [X]Single episode major depression w'out psychotic symptoms |
| Eu32213 | [X]Single episode vital depression w'out psychotic symptoms |
| Eu32300 | [X]Severe depressive episode with psychotic symptoms |
| Eu32311 | [X]Single episode of major depression and psychotic symptoms |
| Eu32313 | [X]Single episode of psychotic depression |
| Eu32400 | [X]Mild depression |
| Eu32500 | [X]Major depression, mild |
| Eu32600 | [X]Major depression, moderately severe |
| Eu32700 | [X]Major depression, severe without psychotic symptoms |
| Eu32800 | [X]Major depression, severe with psychotic symptoms |
| Eu32B00 | [X]Antenatal depression |
| Eu32y11 | [X]Atypical depression |
| Eu32y12 | [X]Single episode of masked depression NOS |
| Eu32z00 | [X]Depressive episode, unspecified |
| Eu32z11 | [X]Depression NOS |
| Eu32z12 | [X]Depressive disorder NOS |
| Eu33.00 | [X]Recurrent depressive disorder |
| Eu33.14 | [X]Seasonal depressive disorder |
| Eu33000 | [X]Recurrent depressive disorder, current episode mild |
| Eu33100 | [X]Recurrent depressive disorder, current episode moderate |
| Eu33200 | [X]Recurr depress disorder cur epi severe without psyc sympt |
| Eu33211 | [X]Endogenous depression without psychotic symptoms |
| Eu33212 | [X]Major depression, recurrent without psychotic symptoms |
| Eu33213 | [X]Manic-depress psychosis,depressd,no psychotic symptoms |
| Eu33214 | [X]Vital depression, recurrent without psychotic symptoms |
| Eu33300 | [X]Recurrent depress disorder cur epi severe with psyc symp |
| Eu33311 | [X]Endogenous depression with psychotic symptoms |
| Eu33312 | [X]Manic-depress psychosis,depressed type+psychotic symptoms |
| Eu33313 | [X]Recurr severe episodes/major depression+psychotic symptom |
| Eu33315 | [X]Recurrent severe episodes of psychotic depression |
| Eu33400 | [X]Recurrent depressive disorder, currently in remission |
| Eu33y00 | [X]Other recurrent depressive disorders |
| Eu33z00 | [X]Recurrent depressive disorder, unspecified |
| Eu33z11 | [X]Monopolar depression NOS |
| Eu34100 | [X]Dysthymia |
| Eu34111 | [X]Depressive neurosis |
| Eu34112 | [X]Depressive personality disorder |
| Eu34113 | [X]Neurotic depression |
| Eu34114 | [X]Persistant anxiety depression |
| Eu3y111 | [X]Recurrent brief depressive episodes |
| Eu41200 | [X]Mixed anxiety and depressive disorder |
| Eu41211 | [X]Mild anxiety depression |
| Eu53011 | [X]Postnatal depression NOS |
| Eu53012 | [X]Postpartum depression NOS |
| ZV11111 | [V]Personal history of manic-depressive psychosis |
| ZV11112 | [V]Personal history of manic-depressive psychosis |

**Anxiety**

| Read code | Description |
| --- | --- |
| 2258 | O/E - anxious |
| 146G.00 | H/O: agoraphobia |
| 1B13.12 | Anxious |
| 1B1V.00 | C/O - panic attack |
| 225J.00 | O/E - panic attack |
| 388w.00 | Generalised anxiety disorder 7 item score |
| E200.00 | Anxiety states |
| E200000 | Anxiety state unspecified |
| E200100 | Panic disorder |
| E200111 | Panic attack |
| E200200 | Generalised anxiety disorder |
| E200300 | Anxiety with depression |
| E200400 | Chronic anxiety |
| E200500 | Recurrent anxiety |
| E200z00 | Anxiety state NOS |
| E202.12 | Phobic anxiety |
| E202100 | Agoraphobia with panic attacks |
| E202200 | Agoraphobia without mention of panic attacks |
| E203.00 | Obsessive-compulsive disorders |
| E203z00 | Obsessive-compulsive disorder NOS |
| E292000 | Separation anxiety disorder |
| E2D0.00 | Disturbance of anxiety and fearfulness childhood/adolescent |
| E2D0000 | Childhood and adolescent overanxiousness disturbance |
| E2D0z00 | Disturbance anxiety and fearfulness childhood/adolescent NOS |
| Eu34114 | [X]Persistant anxiety depression |
| Eu40.00 | [X]Phobic anxiety disorders |
| Eu40000 | [X]Agoraphobia |
| Eu40011 | [X]Agoraphobia without history of panic disorder |
| Eu40012 | [X]Panic disorder with agoraphobia |
| Eu40100 | [X]Social phobias |
| Eu40214 | [X]Simple phobia |
| Eu40y00 | [X]Other phobic anxiety disorders |
| Eu40z00 | [X]Phobic anxiety disorder, unspecified |
| Eu40z11 | [X]Phobia NOS |
| Eu41.00 | [X]Other anxiety disorders |
| Eu41000 | [X]Panic disorder [episodic paroxysmal anxiety] |
| Eu41011 | [X]Panic attack |
| Eu41012 | [X]Panic state |
| Eu41100 | [X]Generalized anxiety disorder |
| Eu41111 | [X]Anxiety neurosis |
| Eu41112 | [X]Anxiety reaction |
| Eu41113 | [X]Anxiety state |
| Eu41200 | [X]Mixed anxiety and depressive disorder |
| Eu41211 | [X]Mild anxiety depression |
| Eu41300 | [X]Other mixed anxiety disorders |
| Eu41y00 | [X]Other specified anxiety disorders |
| Eu41y11 | [X]Anxiety hysteria |
| Eu41z00 | [X]Anxiety disorder, unspecified |
| Eu41z11 | [X]Anxiety NOS |
| Eu42.00 | [X]Obsessive - compulsive disorder |
| Eu42.12 | [X]Obsessive-compulsive neurosis |
| Eu42y00 | [X]Other obsessive-compulsive disorders |
| Eu42z00 | [X]Obsessive-compulsive disorder, unspecified |
| Eu51511 | [X]Dream anxiety disorder |
| Eu93000 | [X]Separation anxiety disorder of childhood |
| Eu93100 | [X]Phobic anxiety disorder of childhood |
| Eu93200 | [X]Social anxiety disorder of childhood |
| Eu93y12 | [X]Childhood overanxious disorder |

**Schizophrenia-related Mental Illness**

| Read code | Description |
| --- | --- |
| 1464 | H/O: schizophrenia |
| 146D.00 | H/O: manic depressive disorder |
| 146H.00 | H/O: psychosis |
| 1S42.00 | Manic mood |
| 212T.00 | Psychosis, schizophrenia + bipolar affective disord resolved |
| 212V.00 | Bipolar affective disorder resolved |
| 212W.00 | Schizophrenia resolved |
| 285..11 | Psychotic condition, insight present |
| 286..11 | Poor insight into psychotic condition |
| E00y.00 | Other senile and presenile organic psychoses |
| E00y.11 | Presbyophrenic psychosis |
| E00z.00 | Senile or presenile psychoses NOS |
| E1...00 | Non-organic psychoses |
| E10..00 | Schizophrenic disorders |
| E100.00 | Simple schizophrenia |
| E100.11 | Schizophrenia simplex |
| E100000 | Unspecified schizophrenia |
| E100100 | Subchronic schizophrenia |
| E100200 | Chronic schizophrenic |
| E100300 | Acute exacerbation of subchronic schizophrenia |
| E100400 | Acute exacerbation of chronic schizophrenia |
| E100500 | Schizophrenia in remission |
| E101.00 | Hebephrenic schizophrenia |
| E101000 | Unspecified hebephrenic schizophrenia |
| E101400 | Acute exacerbation of chronic hebephrenic schizophrenia |
| E101500 | Hebephrenic schizophrenia in remission |
| E101z00 | Hebephrenic schizophrenia NOS |
| E102.00 | Catatonic schizophrenia |
| E102000 | Unspecified catatonic schizophrenia |
| E102100 | Subchronic catatonic schizophrenia |
| E102400 | Acute exacerbation of chronic catatonic schizophrenia |
| E102500 | Catatonic schizophrenia in remission |
| E102z00 | Catatonic schizophrenia NOS |
| E103.00 | Paranoid schizophrenia |
| E103000 | Unspecified paranoid schizophrenia |
| E103100 | Subchronic paranoid schizophrenia |
| E103200 | Chronic paranoid schizophrenia |
| E103300 | Acute exacerbation of subchronic paranoid schizophrenia |
| E103400 | Acute exacerbation of chronic paranoid schizophrenia |
| E103500 | Paranoid schizophrenia in remission |
| E103z00 | Paranoid schizophrenia NOS |
| E104.00 | Acute schizophrenic episode |
| E106.00 | Residual schizophrenia |
| E107.00 | Schizo-affective schizophrenia |
| E107.11 | Cyclic schizophrenia |
| E107000 | Unspecified schizo-affective schizophrenia |
| E107100 | Subchronic schizo-affective schizophrenia |
| E107200 | Chronic schizo-affective schizophrenia |
| E107300 | Acute exacerbation subchronic schizo-affective schizophrenia |
| E107400 | Acute exacerbation of chronic schizo-affective schizophrenia |
| E107500 | Schizo-affective schizophrenia in remission |
| E107z00 | Schizo-affective schizophrenia NOS |
| E10y.00 | Other schizophrenia |
| E10y.11 | Cenesthopathic schizophrenia |
| E10y000 | Atypical schizophrenia |
| E10y100 | Coenesthopathic schizophrenia |
| E10yz00 | Other schizophrenia NOS |
| E10z.00 | Schizophrenia NOS |
| E11..00 | Affective psychoses |
| E11..11 | Bipolar psychoses |
| E11..12 | Depressive psychoses |
| E11..13 | Manic psychoses |
| E110.11 | Hypomanic psychoses |
| E111.00 | Recurrent manic episodes |
| E111000 | Recurrent manic episodes, unspecified |
| E111100 | Recurrent manic episodes, mild |
| E111200 | Recurrent manic episodes, moderate |
| E111300 | Recurrent manic episodes, severe without mention psychosis |
| E111400 | Recurrent manic episodes, severe, with psychosis |
| E111500 | Recurrent manic episodes, partial or unspecified remission |
| E111600 | Recurrent manic episodes, in full remission |
| E111z00 | Recurrent manic episode NOS |
| E113400 | Recurrent major depressive episodes, severe, with psychosis |
| E114.00 | Bipolar affective disorder, currently manic |
| E114.11 | Manic-depressive - now manic |
| E114000 | Bipolar affective disorder, currently manic, unspecified |
| E114100 | Bipolar affective disorder, currently manic, mild |
| E114200 | Bipolar affective disorder, currently manic, moderate |
| E114300 | Bipolar affect disord, currently manic, severe, no psychosis |
| E114400 | Bipolar affect disord, currently manic,severe with psychosis |
| E114500 | Bipolar affect disord,currently manic, part/unspec remission |
| E114600 | Bipolar affective disorder, currently manic, full remission |
| E114z00 | Bipolar affective disorder, currently manic, NOS |
| E115.00 | Bipolar affective disorder, currently depressed |
| E115.11 | Manic-depressive - now depressed |
| E115000 | Bipolar affective disorder, currently depressed, unspecified |
| E115100 | Bipolar affective disorder, currently depressed, mild |
| E115200 | Bipolar affective disorder, currently depressed, moderate |
| E115300 | Bipolar affect disord, now depressed, severe, no psychosis |
| E115400 | Bipolar affect disord, now depressed, severe with psychosis |
| E115500 | Bipolar affect disord, now depressed, part/unspec remission |
| E115600 | Bipolar affective disorder, now depressed, in full remission |
| E115z00 | Bipolar affective disorder, currently depressed, NOS |
| E116.00 | Mixed bipolar affective disorder |
| E116000 | Mixed bipolar affective disorder, unspecified |
| E116100 | Mixed bipolar affective disorder, mild |
| E116200 | Mixed bipolar affective disorder, moderate |
| E116300 | Mixed bipolar affective disorder, severe, without psychosis |
| E116400 | Mixed bipolar affective disorder, severe, with psychosis |
| E116500 | Mixed bipolar affective disorder, partial/unspec remission |
| E116600 | Mixed bipolar affective disorder, in full remission |
| E116z00 | Mixed bipolar affective disorder, NOS |
| E117.00 | Unspecified bipolar affective disorder |
| E117000 | Unspecified bipolar affective disorder, unspecified |
| E117100 | Unspecified bipolar affective disorder, mild |
| E117200 | Unspecified bipolar affective disorder, moderate |
| E117300 | Unspecified bipolar affective disorder, severe, no psychosis |
| E117400 | Unspecified bipolar affective disorder,severe with psychosis |
| E117500 | Unspecified bipolar affect disord, partial/unspec remission |
| E117600 | Unspecified bipolar affective disorder, in full remission |
| E117z00 | Unspecified bipolar affective disorder, NOS |
| E11y.00 | Other and unspecified manic-depressive psychoses |
| E11y000 | Unspecified manic-depressive psychoses |
| E11y100 | Atypical manic disorder |
| E11y300 | Other mixed manic-depressive psychoses |
| E11yz00 | Other and unspecified manic-depressive psychoses NOS |
| E11z.00 | Other and unspecified affective psychoses |
| E11z000 | Unspecified affective psychoses NOS |
| E11zz00 | Other affective psychosis NOS |
| E121.00 | Chronic paranoid psychosis |
| E122.00 | Paraphrenia |
| E12z.00 | Paranoid psychosis NOS |
| E13..00 | Other nonorganic psychoses |
| E134.00 | Psychogenic paranoid psychosis |
| E14..00 | Psychoses with origin in childhood |
| E141.00 | Disintegrative psychosis |
| E141100 | Residual disintegrative psychoses |
| E1y..00 | Other specified non-organic psychoses |
| E1z..00 | Non-organic psychosis NOS |
| Eu02z12 | [X] Presenile psychosis NOS |
| Eu02z15 | [X] Senile psychosis NOS |
| Eu0z.12 | [X]Symptomatic psychosis NOS |
| Eu2..00 | [X]Schizophrenia, schizotypal and delusional disorders |
| Eu20.00 | [X]Schizophrenia |
| Eu20000 | [X]Paranoid schizophrenia |
| Eu20011 | [X]Paraphrenic schizophrenia |
| Eu20100 | [X]Hebephrenic schizophrenia |
| Eu20111 | [X]Disorganised schizophrenia |
| Eu20200 | [X]Catatonic schizophrenia |
| Eu20212 | [X]Schizophrenic catalepsy |
| Eu20213 | [X]Schizophrenic catatonia |
| Eu20214 | [X]Schizophrenic flexibilatis cerea |
| Eu20300 | [X]Undifferentiated schizophrenia |
| Eu20311 | [X]Atypical schizophrenia |
| Eu20400 | [X]Post-schizophrenic depression |
| Eu20500 | [X]Residual schizophrenia |
| Eu20511 | [X]Chronic undifferentiated schizophrenia |
| Eu20y00 | [X]Other schizophrenia |
| Eu20y12 | [X]Schizophreniform disord NOS |
| Eu20y13 | [X]Schizophrenifrm psychos NOS |
| Eu20z00 | [X]Schizophrenia, unspecified |
| Eu21.00 | [X]Schizotypal disorder |
| Eu21.16 | [X]Pseudoneurotic schizophrenia |
| Eu21.17 | [X]Pseudopsychopathic schizophrenia |
| Eu22.00 | [X]Persistent delusional disorders |
| Eu22000 | [X]Delusional disorder |
| Eu22011 | [X]Paranoid psychosis |
| Eu22013 | [X]Paraphrenia - late |
| Eu22100 | [X]Delusional misidentification syndrome |
| Eu22111 | [X]Capgras syndrome |
| Eu22200 | [X]Cotard syndrome |
| Eu22y00 | [X]Other persistent delusional disorders |
| Eu22z00 | [X]Persistent delusional disorder, unspecified |
| Eu23012 | [X]Cycloid psychosis |
| Eu23112 | [X]Cycloid psychosis with symptoms of schizophrenia |
| Eu23214 | [X]Schizophrenic reaction |
| Eu23312 | [X]Psychogenic paranoid psychosis |
| Eu25.00 | [X]Schizoaffective disorders |
| Eu25000 | [X]Schizoaffective disorder, manic type |
| Eu25011 | [X]Schizoaffective psychosis, manic type |
| Eu25012 | [X]Schizophreniform psychosis, manic type |
| Eu25100 | [X]Schizoaffective disorder, depressive type |
| Eu25111 | [X]Schizoaffective psychosis, depressive type |
| Eu25112 | [X]Schizophreniform psychosis, depressive type |
| Eu25200 | [X]Schizoaffective disorder, mixed type |
| Eu25211 | [X]Cyclic schizophrenia |
| Eu25212 | [X]Mixed schizophrenic and affective psychosis |
| Eu25y00 | [X]Other schizoaffective disorders |
| Eu25z00 | [X]Schizoaffective disorder, unspecified |
| Eu25z11 | [X]Schizoaffective psychosis NOS |
| Eu26.00 | [X]Nonorganic psychosis in remission |
| Eu2y.00 | [X]Other nonorganic psychotic disorders |
| Eu2y.11 | [X]Chronic hallucinatory psychosis |
| Eu2z.11 | [X]Psychosis NOS |
| Eu30.00 | [X]Manic episode |
| Eu30.11 | [X]Bipolar disorder, single manic episode |
| Eu30000 | [X]Hypomania |
| Eu30100 | [X]Mania without psychotic symptoms |
| Eu30200 | [X]Mania with psychotic symptoms |
| Eu30211 | [X]Mania with mood-congruent psychotic symptoms |
| Eu30212 | [X]Mania with mood-incongruent psychotic symptoms |
| Eu30y00 | [X]Other manic episodes |
| Eu30z00 | [X]Manic episode, unspecified |
| Eu30z11 | [X]Mania NOS |
| Eu31.00 | [X]Bipolar affective disorder |
| Eu31.11 | [X]Manic-depressive illness |
| Eu31.12 | [X]Manic-depressive psychosis |
| Eu31.13 | [X]Manic-depressive reaction |
| Eu31000 | [X]Bipolar affective disorder, current episode hypomanic |
| Eu31100 | [X]Bipolar affect disorder cur epi manic wout psychotic symp |
| Eu31200 | [X]Bipolar affect disorder cur epi manic with psychotic symp |
| Eu31300 | [X]Bipolar affect disorder cur epi mild or moderate depressn |
| Eu31500 | [X]Bipolar affect dis cur epi severe depres with psyc symp |
| Eu31600 | [X]Bipolar affective disorder, current episode mixed |
| Eu31700 | [X]Bipolar affective disorder, currently in remission |
| Eu31800 | [X]Bipolar affective disorder type I |
| Eu31900 | [X]Bipolar affective disorder type II |
| Eu31911 | [X]Bipolar II disorder |
| Eu31y00 | [X]Other bipolar affective disorders |
| Eu31y11 | [X]Bipolar II disorder |
| Eu31y12 | [X]Recurrent manic episodes |
| Eu31z00 | [X]Bipolar affective disorder, unspecified |
| Eu32300 | [X]Severe depressive episode with psychotic symptoms |
| Eu32800 | [X]Major depression, severe with psychotic symptoms |
| Eu33213 | [X]Manic-depress psychosis,depressd,no psychotic symptoms |
| Eu33312 | [X]Manic-depress psychosis,depressed type+psychotic symptoms |
| Eu33314 | [X]Recurr severe episodes/psychogenic depressive psychosis |
| Eu33315 | [X]Recurrent severe episodes of psychotic depression |
| Eu33316 | [X]Recurrent severe episodes/reactive depressive psychosis |
| Eu3z.11 | [X]Affective psychosis NOS |
| Eu44.14 | [X]Hysterical psychosis |
| Eu84312 | [X]Disintegrative psychosis |
| ZRby100 | Profile of mood states, bipolar |
| ZV11000 | [V]Personal history of schizophrenia |
| ZV11111 | [V]Personal history of manic-depressive psychosis |
| ZV11112 | [V]Personal history of manic-depressive psychosis |
